# The Social Consequences of the Novel Coronavirus Disease (COVID-19) Outbreak in Iran: Is Social Capital at Risk? A Qualitative Study

**DOI:** 10.1155/2021/5553859

**Published:** 2021-06-10

**Authors:** Mohadeseh Motamed-Jahromi, Mohammad Hossein Kaveh

**Affiliations:** ^1^Department of Health Promotion, School of Health, Shiraz University of Medical Sciences, Shiraz, Iran; ^2^Research Center for Health Sciences, Institute of Health, Department of Health Promotion, School of Health, Shiraz University of Medical Sciences, Shiraz, Iran

## Abstract

As well as causing a global health crisis, the COVID-19 pandemic has also generated multilevel social changes by damaging psychosocial and economic resources across Iranian society. Therefore, this qualitative study was conducted to examine and explain these social consequences and their impact on the social capital of Iran during the COVID-19 outbreak. Using a content analysis approach, nine experts participated in semistructured, in-depth interviews. Interviews were recorded and transcribed verbatim and analyzed using Lundman and Graneheim's method. The social impacts of COVID-19 can be summarized into six categories and 32 subcategories. Three positive-negative categories emerged from the data analysis: “formation of new patterns of social communications; formation of new patterns of behavior; creation of economic changes.” Three entirely negative categories included “creating a climate of distrust; disruption of cultural, social, and religious values; psychosocial disorders.” Overall, most findings (27 out of 32 subcategories) indicated the destructive effects of the COVID-19 pandemic on social capital. Therefore, this raises concerns about social capital endangerment in Iran. However, positive social impacts can guide policies that strengthen social action and improve social capital.

## 1. Introduction

Not long after the reporting of a severe respiratory illness in Wuhan, China, in December 2019, the world found itself facing a major health shock, with the disease rapidly spreading to many countries [1]. At the time of writing, 3 million people worldwide have died from COVID-19, and more than 144 million have been infected [2]. Most countries have reported that the disease management and control requirements exceed the existing capacity of their healthcare systems [3]. Therefore, preventive strategies were prioritized to deal with the disease. Compulsory home quarantine was operationalized as a prevention strategy, schools were closed, and many businesses were shut down [4]. Due to this, people's social presence and participation were reduced, and social interactions and mass gatherings that bound people together were limited [5]. In addition, a reduction in the supply of goods and services (including medical devices such as masks and disinfectants) [6], shortcomings in medical services for patients [7], and inconsistencies in socioeconomic and health policies [8] also caused pessimism and reduced trust in governments and organizations. Although the changes in social trends varied from country to country, the continuously destructive effects of this pandemic are extremely worrying. Concurrently, another major concern is the endangerment of social capital.

Social capital is built through social participation, social trust, and trust between government and citizens [9, 10]. It is the glue that connects individuals, families, groups, and community organizations with shared norms, values, attitudes, and beliefs, and it is a valuable resource that plays a key role in health and development [11–13]. Social capital is the mediator between social participation and health, and it provides an opportunity for people to access resources, as well as reducing health inequalities [14, 15]. Therefore, social capital as a national and global wealth is very important to orient human society toward the establishment of human rights and justice [16]. It is noteworthy that social capital is formed over the course of time and that socioeconomic and political conditions at the national and international levels have a tremendous impact on it [17, 18]. Therefore, it seems necessary to study the situation of social capital during the COVID-19 pandemic in countries involved in various social, economic, and political crises, such as Iran.

Iran is a developing country, and the COVID-19 pandemic occurred in the midst of an existing economic crisis in the country. International sanctions against Iran slowed economic growth rates (-4.99%) and increased inflation rates (30.52%) in 2020 [19]. Therefore, the Iranian people's ability to fight COVID-19 has been severely weakened [20]. This economic situation has led to a lack of funding allocated for the prevention, diagnosis, and treatment of COVID-19, and the healthcare system cannot take appropriate measures to purchase vaccines, drugs, and full coverage of health insurance [21]. While essential medicines and medical devices are spared from sanctions, sanctions have limited their accessibility by reducing production capacity, as well as adding to problems of trade and foreign exchange [22]. In addition, the sharp rise in drug prices has affected approximately 6 million patients with complex and chronic diseases, exposing them to disease progression and a high risk of infections such as COVID-19 [22, 23]. It seems to be causing rising frustration with and distrust of social and governmental institutions at this turbulent time, and the short-term and long-term social consequences of the COVID-19 outbreak are emerging in Iranian society. Inasmuch as these consequences can devastate the social capital of the Iranian population, further research on the effects and consequences of the COVID-19 pandemic is necessary in order to carry out social development policy and planning effectively.

The social effects of past epidemics have been variously considered in previous studies. It should be noted that the main focus of these studies has not been on social capital; its positive role in improving prevention measures in society has been more greatly emphasized. A review of studies shows that, at the time of the Ebola outbreak, strong leadership and the strengthening of social capital were among the factors that strengthened bonds in society and trust in the health system, which was helpful in alleviating the shock of Ebola [24]. A study in Taiwan indicated that social capital helped to adopt preventive and health-oriented behaviors during an influenza pandemic [25]. Pattussi et al. came to the conclusion that increasing social capital reduces stress and mental illness and increases health-related behaviors [26]. Seddighi et al. stated in a report that strengthening social capital in Iran can unite people, the government, and the private sector in a response to a disaster such as the COVID-19 pandemic [27].

A search of PubMed, Science Direct, and Google Scholar databases until May 15, 2020, indicated no study that examined the social effects of the COVID-19 pandemic via a qualitative approach in Iran. Since the social consequences of COVID-19 are wider and more persistent than previous epidemics, and on the other hand, tools for quantitative study have not yet been provided, this qualitative study aimed to explore the social implications of COVID-19 in Iran, especially the status of social capital.

## 2. Materials and Methods

This study was carried out using a qualitative content analysis approach in order to explore the views of experts and faculty members from different fields affiliated with the Shiraz University of Medical Sciences, with reference to the social consequences of the COVID-19 outbreak in Iran. Shiraz University of Medical Sciences, the second-largest university in Iran with a total of 14 faculties, 780 faculty members, 10,000 students, 11 hospitals, and 50 healthcare centers, thus can be considered a good setting to conduct this study. This research was reported according to the Standards for Reporting Qualitative Research (SRQR) [28].

A purposive sampling method with maximum variation was used to recruit the experts. They were identified based on the diversity of age, gender, fields of study, and scientific rank. Inclusion criteria were being a faculty member in different schools affiliated with the University of Medical Sciences, having at least three years of work experience and participating in the study. Exclusion criteria included refusal to participate in the interview and dissatisfaction with the interview recording. In this study, semistructured and in-depth interviews were used as data collection strategies. The face-to-face interviews were conducted at participants' workplaces according to their preferences. The first author, a doctoral student in health promotion, conducted the interviews and then transcribed them verbatim (M. MJ). The second author, an associate professor of health promotion, reviewed and audited the interview process (MH. K). Both authors have been actively involved in data analysis and the extraction of code and categories.

During the interview, participants first answered an open-ended question: “What do you think are the social consequences of COVID-19 prevalence?” A following probing question was also used to clarify the participants' answers: “Could you please explain more about your response?” In total, each interview lasted approximately 45 minutes, and participants gave their permission to record audio. Field notes were taken, and interviews were audio-recorded and transcribed verbatim. Data analysis was performed immediately after each interview. Interviews were continued until operational and theoretical data saturation. Operational data saturation indicated that most codes were obtained in the first interviews, and the number of new codes had a decreasing trend in subsequent interviews [29]. Theoretical data saturation was a point where the researchers did not obtain any new codes or concepts via the iterative process in their meetings [30]. Lundman and Graneheim's five-step content analysis method was applied for data analysis [31].

Guba and Lincoln's criteria, known as credibility, dependability, transferability, and confirmability, were utilized to establish trustworthiness [32]. Credibility creates confidence that the findings are accurate, reliable, and acceptable from the participants' viewpoint [33]. In this study, credibility was enhanced by field note-taking, peer debriefing sessions with two experts who were not involved in the research project, and interviewers' engagement with participants and the research context for several weeks [34–36]. Dependability confirms that the findings are repeatable if the study is performed in the same setting, and was established by checking the written transcripts against the audio-recorded data for accuracy and rich explanation of research methods [33]. Confirmability shows the degree to which other researchers would confirm findings, and it is ensured by using a separate reflexive journal for each researcher, then discussing them in daily meetings [37]. Transferability refers to the probability that findings can be generalized to other contexts or settings [33]. Purposive sampling with maximum diversity and operational and theoretical data saturation increased the data transferability [34, 38, 39].

The present study is the result of an approved research project at the University of Medical Sciences. In this study, ethical and fiduciary principles were observed in the use of resources and data collection. Written informed consent was obtained for the interview and its recording, and no compulsion was applied to participants to continue in the study. The confidentiality of the information was also taken into account.

## 3. Results

**Figure 1:**
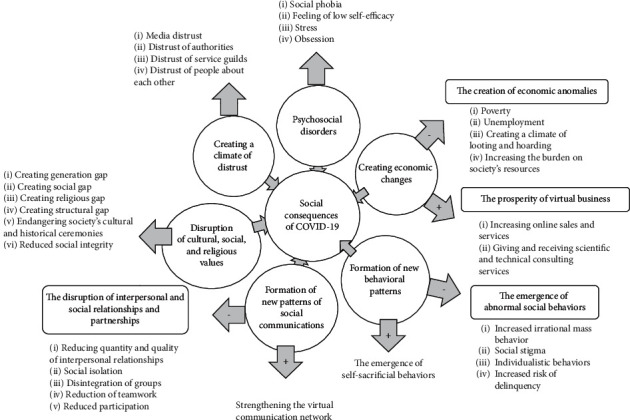
Categories and subcategories that highlight the social consequences of the COVID-19 outbreak.

In the present study, nine experts' views on the social consequences of the COVID-19 outbreak were explored (Table 1). Based on the data analysis, 167 initial codes were obtained, after which the reading was reduced to 58 codes; of these, 32 subcategories were extracted, and finally, six categories were obtained, including three positive-negative categories and three completely negative categories (Figure 1).

### 3.1. Formation of New Patterns of Social Communication

This category is divided into positive and negative aspects of new communication patterns. The positive side involves strengthening the virtual communication network, and the negative aspect is the disruption of interpersonal and social relationships and partnerships.

#### 3.1.1. Strengthening the Virtual Communication Network

The COVID-19 outbreak drew people's attention to the capacities of information and communication technology. People increasingly used these technologies, especially social media, for social interaction and economic transactions. One of the participants said:

“…In the Corona epidemic, staying at home led to more use of cyberspace, and now the source of information for Iranian families has become virtual communication networks...” (P2)

#### 3.1.2. The Disruption of Interpersonal and Social Relationships and Partnerships

According to most interviewees, this epidemic has reduced the quantity and quality of interpersonal relationships. Social isolation, disintegration of groups, reduction of participation, and teamwork reduction are other factors that have damaged the communication network.

“… the result of this situation is the reduction of people's presence in society, reduction of their social participation, and even disintegration of social groups such as the elderly or young people groups in a neighborhood...” (P1)

### 3.2. Formation of New Behavioral Patterns

This category consists of two parts: the emergence of abnormal social behaviors, which is a negative aspect of behaviors, and a positive aspect, the emergence of self-sacrificial behaviors. Experts stated that increased irrational mass behavior, social stigma, individualistic behaviors, and increased risk of delinquency are abnormal social behaviors.

“…We're getting to a point which probably creates a group excitement toward shopping, patients, quarantined locations, and so on.” (P8)

“…you can walk 100 meters from the college, and more than a hundred disposable gloves and paper napkins have been dropped without any protection...” (P6)

“… consider a person whose job is a food driver, and restaurants have been closed these days. This person, who owns only a motorcycle, has no other choice but to commit delinquency...” (P7)

The emergence of self-sacrificing behaviors is another new pattern of behavior that occurred during the COVID-19 outbreak.

“…I recently saw a clip of a woman in Lorestan taking a bottle of alcohol and a cloth in her hand and disinfecting an ATM near her place of residence...” (P2)

### 3.3. Creation of Economic Changes

This category also includes two parts: its positive part is the prosperity of virtual business and its negative aspect is the creation of economic anomalies.

#### 3.3.1. The Prosperity of Virtual Businesses

Increasing online sales and services, as well as giving and receiving scientific and technical consulting services, is a sign of virtual business prosperity.

  “…because of being at home, the amount of online shopping has increased, and banking transactions are done through mobile apps...” (P1)
  “…These days, people prefer to use online and telephone counseling services in various fields of education, technology, health...” (P3)

#### 3.3.2. The Creation of Economic Anomalies

Poverty, unemployment, increasing the burden on society's resources, and creating a climate of looting and hoarding are among the economic anomalies created by the COVID-19 outbreak.

“…People are afraid to buy; they are afraid of getting infected. It has caused a lot of damage, especially to middle-class people...” (P5)

  “…What is going to happen to those who are unemployed and have no economic support?” (P8)

“…We are faced with an unreasonable demand of bulk-buying toilet paper, masks, soap, and disinfectant. Undoubtedly, the government will be in trouble providing hospital space and medical supplies...” (P8)

### 3.4. Creation of a Climate of Distrust

According to experts, one of the social effects of the COVID-19 outbreak is creating a climate of mistrust among citizens. They acknowledged that this climate of distrust is due to distrust of authorities' honesty, mass media, service guilds, and skepticism of people toward each other.

  “…I think the creation of a climate of uncertainty among the people is because of uncertainty toward the officials...” (P6)
  “…People distrust mass media because of internal media's dishonesty and foreign media's misrepresentation ...” (P3)

Participant No. 1 also believed:

“…there are uncertainty and suspicion in the interactions between people and organizations and service units. For example, people are skeptical about whether they regularly disinfect the restaurant's work environment and tools...”

### 3.5. Disruption of Cultural, Social, and Religious Values

From the experts' points of view, some of the problems following the COVID-19 outbreak are creating generational, social, religious, and structural gaps, as well as the disruption of society's cultural and historical ceremonies, which can cause reducing social convergence.

“…only doubt about a person's sickness may be an excuse to reduce contact with her or him, which is harmful to social convergence and social integrity...” (P8)

Participant No. 8 also said about the generation gap:

“…These days, the parental obsession towards hygiene adherence, and the characteristics of teenagers who don't feel afraid of anything, can cause parent-and-child challenges...”

Another participant mentioned an example of a religious gap:

“…Some religious people agree that people should currently leave religious mass ceremonies based on scientific reasons. Other religious groups insist on holding mass ceremonies.” (P7)

And an example of a structural gap is that:

“…in parliament, a bag of gloves and disinfectants was given to the deputies, while at the same time, the people did not have these supplies; I think this caused a deeper gap between the officials and the people...” (P9)

Another concern of experts is the disruption of the cultural and historical ceremonies. As participant No. 4 said:

“…because of Corona, we do not have the excitement of the New Year, we do not have family gatherings, and we do not have goldfish on the Eid-e Nowruz table...”

### 3.6. Psychosocial Disorders

According to experts, social phobia, stress, low self-efficacy, and obsession are psychosocial disorders that people experience following the COVID-19 epidemic.

“…When a disease spreads, people experience generalized anxiety and phobia. One of the reasons is that people are afraid about who should take care of them well…” (P2)

“…feelings of inefficiency are one of the psychological consequences due to the contradiction of the messages. Obsession can also be caused by overuse of disinfectants, gloves, and masks...” (P7)

## 4. Discussion

The current study results showed a range of positive and negative social consequences of the COVID-19 outbreak. In comparison, the frequency of negative and destructive outcomes (27 out of a total of 32 subcategories) outweighs the positive outcomes. The higher proportion of this disease's negative consequences also raises doubts about the endangerment of social capital [40]. This issue will be explored below with reference to pertinent literature and evidence, providing a deeper analysis of the findings.

According to the results, the COVID-19 epidemic has caused damage to social networks and interpersonal relationships and reduced participation and teamwork in Iranian society. Since social capital refers to aspects of social structure that facilitate engagement and collaboration for individual mutual benefit and achievement to group goals [41–43], it therefore seems that social dysfunction will damage social capital during the COVID-19 outbreak. In line with this finding, Hossini Rafsanjanipoor et al. concluded that the COVID-19 outbreak has caused social dysfunction in the majority of the Iranian population, and appropriate interventions are necessary to deal with these problems [44]. Since mosques are places where the Muslim people of Iran gather daily and interact and cooperate with each other, it is recommended that virtual platforms be used to connect the people of each neighborhood through the custodians of mosques during the current situation. Virtual communication and cooperation to solve the living problems of the poor and elderly can strengthen social capital to some extent and make it easier to endure economic problems and psychosocial damage.

The formation of new patterns of behavior is also one of the most interesting social consequences of the COVID-19 outbreak. Self-sacrificial behaviors are an example of the changing valuable behaviors that Iranian society has witnessed. On the other hand, abnormal social behaviors, such as increased irrational mass behaviors, social stigma, individualistic behaviors, and increased delinquency, have also been more prevalent following the outbreak of COVID-19 in Iran and have caused damage to social capital. Ling and Ho reached the same conclusion that, during the COVID-19 pandemic, most people behave selfishly and opportunistically to gain maximum personal benefit, even if it endangers others [45]. When people lose feelings of mutual responsibility and social cohesion and become selfish, it leads to social costs, reduced collaboration, and, eventually, the erosion of social capital [46, 47]. The positive behavioral values of the Iranian people can be refreshed through cultural programs through the mass media, as well as remembering the Persian proverb: “Human beings are members of a whole, in the creation of one essence and soul.” Such reminders can also reduce selfish and harmful behaviors.

Positive economic changes, such as the prosperity of virtual business in the form of online sales and services and giving and receiving scientific and technical consulting services, were able to improve the financial situation of some Iranian people during the economic crisis and the COVID-19 outbreak. On the other hand, however, the majority of Iranian people are caught in a circle of poverty and unemployment, and simultaneously looting, hoarding, and panic-buying have caused a decrease in public trust and social cohesion. Since the components of social capital, i.e., trust, relationships, and social networks, can be transformed into economic capital [48], social capital will also be damaged if there is an abnormality in economic capital. Charitable donations to the poor are a temporary solution to this problem. Politicians must act as soon as possible, however, to lift international sanctions so that, with an economic boom, Iranian society can more easily fight COVID-19.

One of the findings of this study is the emergence of a climate of mistrust following the COVID-19 epidemic in Iran, which occurred due to a lack of transparency and ill-considered strategies in the government and mass media. Trust in governments' honesty and interpersonal networks plays an essential role in building social capital and in encouraging health protection behaviors among people [13, 25, 49, 50]. Therefore, violating public trust leads to the erosion of social capital. In line with this finding, Fang et al. stated that Taiwanese people did not care about government messages, due to uncertainty and doubt during the influenza pandemic [51]. Since the Iranian people have always supported the government in times of difficulty, the best way to overcome these problems is in the honesty of the government and the mass media and the transparency of their actions. Government officials also need to build trust among their citizens long before crises and epidemics strike so that their precautionary messages will be accepted by the people [26].

According to the findings, the generational, social, structural, and religious gaps and disruption of society's cultural and historical ceremonies are also among the social consequences of the COVID-19 outbreak, which has led to the breakdown of sociocultural values. Different risk perceptions of COVID-19 between younger and older Iranian people, as well as some religious people's opposition to the cancellation of ceremonies and gatherings, have resulted in reduced solidarity and social support and eventually in damaged social capital. Likewise, the inequitable availability of more facilities for the officials and social conflict between the tribes and communities have also added to these deleterious consequences. Several researchers, in accordance with this finding, have stated that damage to social capital is due to the damage done to reciprocal norms, values, and attitudes that establish the importance of citizenship, civilization, and civic ethics [41, 52]. Culture-building and education, convincing religious leaders and tribal leaders, and eliminating discrimination and injustice can be among the solutions to these problems in Iran's diverse society, in terms of both ethnicity and social class.

Social phobia and stress following COVID-19 are above the Iranian people's tolerance level, as they have already faced high levels of pressure due to international sanctions, inappropriate policies, and political and social crises over the previous year. These crises have disrupted society's mental structure and caused reductions in people's self-efficiency and self-control. Actually, decreasing resilience, adaptation, and social support in a community can lead to social isolation, reduced participation, and finally decreased social capital [53]. To overcome these simultaneous crises, the problems of the Iranian people must be addressed radically. Therefore, the most important policy should be to try to lift international sanctions. It is also necessary to take basic measures to create psychological and social security and to form committees to provide psychological support to the people through governmental and nonprofit organizations.

## 5. Conclusion

Overall, this study concludes that the COVID-19 outbreak has a variety of social consequences. A reflection on the higher rates of negative outcomes and social capital components indicates that the COVID-19 outbreak could jeopardize the social capital of the Iranian people. Of course, factors such as cultural differences, social and organizational capacities, management and leadership patterns, and social media's performance can moderate this epidemic's effects on social capital during the time. It is possible to address these challenges and problems through continuous training and acculturation, transparency of organizations, increasing trust, and a sense of belonging to the community. Considering the unfavorable social results of the COVID-19 disease outbreak in Iran and its destructive effects on social capital, it is recommended that a comprehensive document of realistic solutions should be developed and implemented with the participation and cooperation of officials and healthcare providers. Positive social impacts can guide policies that strengthen social action and, consequently, improve social capital. It is also suggested that the impact of the social outcomes of the COVID-19 outbreak on social capital should be determined, in order to adopt correct preventive policies and strategies and to improve social development processes. The overall findings of this study (though not all of them) seem to be relatively generalizable to developing countries in conflict or crisis situations. However, more accurate judgments will require additional comparative research.

There may be limitations to the generalizability of these findings, as the social and economic conditions of Iran at present are unique and may differ from other countries. In addition, the participants were not selected randomly, which can limit the scope of the generalizability of the findings.

## Figures and Tables

**Table 1:** Demographic data of interview participants.

Participant number	Gender	Age	Professional background	Years of experience
1	Male	53	Health promotion	27 years
2	Male	42	Sociology	5 years
3	Female	43	Health promotion	11 years
4	Male	35	Public health	6 years
5	Male	56	Occupational health	28 years
6	Male	41	Health economics	8 years
7	Male	42	Physician	5 years
8	Female	43	Psychology	15 years
9	Male	47	Sociology	12 years

## Data Availability

The data used to support the findings of this study are restricted by the Ethics Committee of Shiraz University of Medical Sciences in order to protect participant privacy. Data without names are available for researchers who meet the criteria for access to confidential data.
